# Smart Device for Biologically Enhanced Functional Regeneration of Osteo–Tendon Interface

**DOI:** 10.3390/pharmaceutics13121996

**Published:** 2021-11-24

**Authors:** Angela Faccendini, Eleonora Bianchi, Marco Ruggeri, Barbara Vigani, Cesare Perotti, Francesco Claudio Pavesi, Laura Caliogna, Francesca Natali, Elena Del Favero, Laura Cantu’, Franca Ferrari, Silvia Rossi, Giuseppina Sandri

**Affiliations:** 1 Department of Drug Sciences, University of Pavia, Viale Taramelli 12, 27100 Pavia, Italy; angela.faccendini@gmail.com (A.F.); eleonora.bianchi04@universitadipavia.it (E.B.); marco.ruggeri02@universitadipavia.it (M.R.); barbara.vigani@unipv.it (B.V.); franca.ferrari@unipv.it (F.F.); silvia.rossi@unipv.it (S.R.); 2 Immunohaematology and Transfusion Service, Apheresis and Cell Therapy Unit, Fondazione IRCCS Policlinico San Matteo, 27100 Pavia, Italy; c.perotti@smatteo.pv.it; 3 Orthopedy, Fondazione IRCCS Policlinico San Matteo, 27100 Pavia, Italy; claudiopavesi@tiscali.it (F.C.P.); l.caliogna@smatteo.pv.it (L.C.); 4 Institut Laue-Langevin, 71 Avenue des Martyrs, CS 20156, CEDEX 09, 38042 Grenoble, France; natali@ill.fr; 5 Department of Medical Biotechnology and Translational Medicine, University of Milan, LITA Viale Fratelli Cervi 93, 20090 Segrate, Italy; elena.delfavero@unimi.it (E.D.F.); laura.cantu@unimi.it (L.C.)

**Keywords:** electrospinning, tubular structure, hybrid scaffold, platelet lysate, hydroxyapatite, polysaccharides

## Abstract

The spontaneous healing of a tendon laceration results in the formation of scar tissue, which has lower functionality than the original tissue. Moreover, chronic non-healing tendon injuries frequently require surgical treatment. Several types of scaffolds have been developed using the tissue engineering approach, to complement surgical procedures and to enhance the healing process at the injured site. In this work, an electrospun hybrid tubular scaffold was designed to mimic tissue fibrous arrangement and extracellular matrix (ECM) composition, and to be extemporaneously loaded into the inner cavity with human platelet lysate (PL), with the aim of leading to complete post-surgery functional regeneration of the tissue for functional regeneration of the osteo–tendon interface. For this purpose, pullulan (P)/chitosan (CH) based polymer solutions were enriched with hydroxyapatite nanoparticles (HP) and electrospun. The nanofibers were collected vertically along the length of the scaffold to mimic the fascicle direction of the tendon tissue. The scaffold obtained showed tendon-like mechanical performance, depending on HP content and tube size. The PL proteins were able to cross the scaffold wall, and in vitro studies have demonstrated that tenocytes and osteoblasts are able to adhere to and proliferate onto the scaffold in the presence of PL; moreover, they were also able to produce either collagen or sialoproteins, respectively—important components of ECM. These results suggest that HP and PL have a synergic effect, endorsing PL-loaded HP-doped aligned tubular scaffolds as an effective strategy to support new tissue formation in tendon-to-bone interface regeneration.

## 1. Introduction

Tendon pathologies are medical conditions, and include ruptures and overuse injuries with inflammatory and degenerative alterations, such as tendinopathies [1]. In the United States, 33 million musculoskeletal injuries have been reported per year, 50% involving tendons and ligaments. Tendon injuries are common in physically active people, both adult and young (especially male), as well as in sedentary population with moderate physical activity. Moreover, several intrinsic factors, including body weight, nutrition, age, and genetic diseases that affect the connective tissue, could have an impact on tendon/ligament integrity. The incidence of Achilles tendon ruptures (one of the most frequently injured tendons), is up to 1%, and typically in 30–50-year-old men [2].

Chronic, nonhealing tendon injuries frequently require surgical treatment, and, despite recent advancements in orthopaedic surgery, current tendon repair techniques yield less-than-optimal results [3,4,5]. Moreover, healed tendons tend to form scar tissue with mechanical properties different as compared to healthy ones, and are prone to reinjury.

Recently, several approaches have been proposed to complement surgical procedures to enhance the healing process at the injured site. In particular, growth factors (GFs) have been proven effective, especially in the early phases of healing process. Among them, transforming growth factor beta (TGF-β), epidermal growth factor (EGF), platelet derived growth factor (PDGF-B), fibroblast growth factor (bFGF), vascular endothelial growth factor (VEGF), and hepatocyte growth factor (HGF) have been identified as key factors [6]. The hemoderivative human platelet lysate (PL) is rich in GFs and has been widely explored for treatment of tendon injuries since the 1980s. Nonetheless, despite the numerous positive reports on the use of hemoderivatives, some inconsistent and sometimes contradictory results are still frequently found. This seems mainly related to the difficulties in obtaining a suitable release of GFs for hemoderivatives as for time, dose, site, and target. A proper hosting biomaterial platform could help in overcoming these limitations and should enable a controlled spatiotemporal and selective delivery of signaling biomolecules. In a biomimetic regenerative medicine approach, this should potentiate the therapeutic effect and foster tissue regeneration [7]. 

The aim of this work was the design and the development of 3D scaffolds as orthopaedic surgical supports, able to achieve time-, site-, target-, and dose dependent release of GFs to promote tendon and tendon-to-bone interface functional regeneration.

The 3D scaffolds were assembled to mimic the hierarchical arrangement of tendon collagen bundles. For this purpose, hollow tubular 3D scaffolds were formed by electrospinning of nanofibers with preferential axial alignment: this topography was intended to guide cell migration and orientation and facilitate remodeling in the last phase of the healing process [8]. In fact, the alignment of the nanofibers in scaffolds has been reported as a crucial point to favor tendon cell differentiation by transducing biochemical signals since cells are capable of sensing the geometry of fiber surface [9]. Moreover, the hollow tubular structure was intended to achieve a spatiotemporal controlled release of GFs by loading PL into the inner cavity of the scaffold. In fact, PL has been reported to be highly effective for enhancing tendon and osteoblasts growth [10,11]. 

The 3D scaffolds were based on polysaccharides biopolymers, in particular, chitosan, pullulan, and chondroitin sulfate [12,13,14].

To further improve healing [15,16], the doping of nanofibers with hydroxyapatite (HP), a bioceramic material mimicking the mineral content of extracellular matrix in the bone, was also considered. In fact, HP has been recognized as an active agent capable of supporting soft and hard tissue reparation, thanks to its biomimetic cue, and restoring tendon-to-bone insertion [6,15,17,18,19,20]. From this perspective, nano-bioceramic/biopolymer composites have been identified as an innovative and powerful tool to improve the interaction between scaffolds and host cells in interface bio-engineering [15,16]. Moreover, the doping of HP nanoparticles into the polymeric matrix could tune the scaffold porosity and mechanical properties, their degradation in vivo, and the intracellular signaling response, creating a smart composite biomaterial [16,21].

The 3D tubular scaffolds, formulated according to the mentioned aims, were characterized for morphology, composition, and mechanical properties. Moreover, biocompatibility and effectiveness were assessed in vitro, towards both tenocytes (normal human tenocytes, TEN-1) and osteoblasts (SAOS-2 cells). The effectiveness of PL to further enhance cell proliferation and production of extracellular matrix was evaluated. Finally, the effect of combining nanobioceramic/biopolymer hybrid scaffolds with PL (rich in GFs), to provide a synergic platform for cellular growth characterized by suitable morphology and extracellular-like environment was also assessed. 

## 2. Materials and Methods

### 2.1. Materials

3D scaffolds: chitosan (CH), deacetylation degree 98%, MW 251000 Da, (ChitoClear, Iceland); pullulan (P), food grade (Hayashibara, Okayama, Japan); citric acid (CA) (Carlo Erba Reagents, Cornaredo (MI), Italy); hydroxyapatite (HP), nanopowder < 200 nm particle size, ≥97% synthetic (Sigma-Aldrich, St. Louis, MO, USA).

Platelet lysate (PL): PL was obtained from the Apheresis Service of Immunohematology and Transfusion Service Centre for transplant immunology, by employing a sterile connection technique. Aliquots of hyper-concentrate platelets (high platelet concentration in small plasma volume and minimal leukocyte contamination) were obtained from apheresis, carried out on regular blood donors (Immunohematology and Transfusion Service, Apheresis and Cell Therapy Unit, Fondazione IRCCS Policlinico S. Matteo, Pavia, Italy). The platelet pool was frozen at −80 °C for 5 h and subsequently unfrozen in a sterile water bath at 37 °C. An automated platelet count and tests for aerobic, anaerobic and fungi contamination were performed after saline dilution.

### 2.2. Methods

#### 2.2.1. Preparation of the Polymeric Blends

The polymeric blend was based on CH and P; 20% (*w*/*w*) P was dissolved in water, while 5% (*w*/*w*) CH and 5% (*w*/*w*) CA were both solubilized in acetic acid:water (90:10) [14]. The polymeric blend was prepared by mixing a 1:1 ratio the two polymeric solutions previously prepared under magnetic stirring, at room temperature, to obtain the blank formulation (B). The 0.1 HP and 0.5 HP formulations were prepared by adding HP nanoparticles to the final polymeric blend, 1 h before electrospinning, to obtain 0.1% and 0.5% (*w*/*w*) final mineral concentrations, respectively.

#### 2.2.2. Preparation of Electrospun Scaffolds

Scaffolds were obtained from the B,0.1 HP and 0.5 HP blends using an electrospinning apparatus (STKIT-40, Linari Engineering, Pisa, Italy) equipped with a high-voltage power supply (Razel R99-E 40 kV), a 10 mL syringe with inox 21G needle, and a volumetric pump (Razel R99-E). A static and flat collector was used to obtain the random scaffolds (namely, R-B, R-0.1 HP, and R-0.5 HP). The lengthwise-aligned tubular scaffolds (namely, A-B, A-0.1 HP, and A-0.5 HP) were collected in a tubular shape using a cylindrical rotating drum in stainless steel (dimensions: diameter: 3 mm; length: 150 mm; wall thickness: 0.3 mm). Finally, all the electrospun scaffolds were crosslinked by dry heating at 150 °C for 1 h [22]. This process is also reported as appropriate for sterilizing the products. In Table 1, the compositions of the scaffolds prepared are reported.

#### 2.2.3. Scaffold Chemical–Physical Characterization

TEM analysis (JEOL JEM-1200 EX II microscope; CCD camera Olympus Mega View G2 with 1376 × 1032 pixel format, Tokyo, Japan) was performed to assess the HP inclusion in the electrospun nanofibrous structure (operating HV at 100 kV; magnifications: 15k, 25k, 50k). For this purpose, a thin layer of fiber was electrospun directly onto the TEM grids (formavar/carbon 300 mesh Cu, Agar Scientific, Monterotondo (RM), Italy) and cross-linked by heating, as previously described before the analysis.

Scaffold morphology was characterized by means of SEM analysis (Tescan, Mira3XMU, platinum sputtering, Brno, Czech Republich). Electrospun nanofiber diameters, pore size, and degree of orientation (alignment) were assessed by image analysis software (ImageJ, ICY, Institut Pasteur, Paris, France). 

The wettability of the electrospun fibers was assessed with a contact angle meter (DMe-211 Plus; FAMAS software, Kyowa, Osaka, Japan). The droplet shape (0.4 µL of PBS) was captured through the CCD camera at 1 s after the droplet touched the scaffold interface. 

FT-IR analysis was carried out by means of an infrared imaging microscope (FT-IR, Spectrum BX, Perkin Elmer, Milano, Italy). The infrared spectra were acquired in the range 4000–400 cm^−1^. The measurements were performed on cutouts of the random crosslinked scaffolds (R-B, R-0.1 HP, R-0.5 HP), 5 × 5 mm^2^, 10 µm thick.

#### 2.2.4. Scaffold Mechanical Properties

Scaffolds were subjected to tensile measurements using a TA.XT plus apparatus (Stable Microsystems, Venezia, Italy), equipped with a measurement system A/TG (5 kg loading cell) formed by two grips, one fixed and the other movable. The random scaffolds were cut to obtain a 3 cm long × 1 cm wide specimen (exposed area for the test: 1 × 1 cm^2^). For the tubular aligned scaffolds, 2 cm long × 0.5 cm wide two-folded pieces were cut (unfolded width: 1 cm). Each sample was then placed between the texture grips, so that the exposed area for the test was the same for all: 1 × 1 cm^2^. The movable grip was moved forward at 0.5 mm/s until sample breakdown, up to 3 cm.

The force at break (the load-to-failure), maximum tensile force (Fmax, Pa) was normalized for thickness and area between the grips. The elongation values (E%) were obtained as follows:

E% = 100 × (L_fin_ − L_in_)/L_in_

where L_in_ and L_fin_ are the distances between the grips at the beginning and at scaffold break.

The Young modulus (YM) was calculated as the slope of Fmax vs. grip displacement, in the linear region. The mechanical properties were investigated in the dry and hydrated states. The latter was prepared by spraying milliQ water at 37 °C on the scaffold surface 30 s before testing [23,24].

#### 2.2.5. Local Dynamics in Scaffolds

The B and 0.1 HP scaffolds in the random structure, in the dry and D_2_O or H_2_O hydrated conditions, were submitted to neutron scattering measurements to test for local mobility and rigidity, on the molecular scale. Rectangular weighted cuts (3 × 5 cm^2^) of the scaffolds were enclosed in air-tight aluminum flat cells for measurement. Experiments were performed on the thermal (λ = 2.23 Å) high-energy resolution backscattering spectrometer IN13 (Institut Laue-Langevin, ILL, Grenoble, France), in the temperature range 280 < T < 315 K. The elastic neutron scattering intensity, S(q, ω = 0), decreases as a function of the scattering angle θ, with a behavior that, at low angles, has a typical decay constant proportional to the mean-square amplitude of atomic displacements (MSD). The variation of the MSD as temperature is raised can be interpreted in terms of an empirical effective force constant <k′>, called resilience by Zaccai [25], which describes the rigidity, or resilience, of the macromolecules on the local scale (see also Appendix A).

#### 2.2.6. Platelet Lysate Release

0.2 cm^2^ scaffolds (5 mm diameters 0.2 mm thickness) were placed onto inserts in a 96-well plate (4.26 mm insert diameter; 3 μm membrane pore diameter, Corning Transwell Cell Culture Plate, Merck, Sigma Aldrich, Italy) to perfectly cover them; 70 µL HBSS (with CaCl_2_, MgCl_2_, Gibco, Thermofisher, Monza, Italy) were added in the apical chamber and 200 µL PL (1:10 dilution in HBSS) were placed in the basal one to simulate PL loading into the void of the tubular scaffold. At fixed times, a 50 µL aliquot was withdrawn from the apical phase and replaced with fresh HBSS. In a separate experiment, diffusion of PL proteins through the insert was verified to be quantitative and completed within 12 h.

The samples obtained at different timing were analyzed using Bradford assay (Bradford Reagent, Sigma-Aldrich, Milano, Italy) to assess the total amount of proteins permeated across the wall; 5 µL of each sample was admixed with 250 µL of the reagent (0.045% Brilliant Blue G in 4% methanol plus 10% phosphoric acid) directly in a well of a 96-well plate (Sigma-Aldrich, Milano, Italy). After stirring and incubation at room temperature for 30 min, their absorbance was measured at 595 nm wavelength, using an ELISA plate reader (Biorad, Segrate (MI), Italy).

Albumin was used as a model protein and the calibration curve was linear in the concentration range 0.131–1.4 mg/mL with R > 0.995. The percent of protein content of the tubular scaffold at different timing along the release process was calculated as

%P_scaff(t)_ = 100 × (P_tot_ − P_rel(t)_)/P_tot_

where P_scaff(t)_: percentage amount of proteins permeated at time t; P_tot_: total amount of proteins in the basal compartment; P_rel(t)_: amount of proteins assayed in the apical compartment.

#### 2.2.7. Cytocompatibility, Adhesion, and Proliferation Assay: TEN-1 and SAOS-2 Cell Cultures

Cytocompatibility, adhesion and proliferation assays were carried out using two cell types: normal human tenocytes (TEN-1) (passages number 1–5, ZenBio, Durham, NC, USA) and SAOS-2 (human osteogenic sarcoma, Sigma-Aldrich, Milano, Italy).

TEN-1 were cultured in collagen (rat tail collagen coating solution, Cell Applications, Italy) coated flasks, using tenocyte growth medium, (ZenBio, USA). SAOS-2 cells were cultured using McCoy (McCoy’s 5A Medium Modified, with L-Glutamine and sodium bicarbonate, Sigma-Aldrich, Milan, Italy) supplemented with 1% *v*/*v* penicillin-streptomycin-amphotericin (pen/strep/ampho100x, Euroclone, Pero (MI), Italy) and 10% *v*/*v* FBS (fetal bovine serum, Euroclone, Pero (MI), Italy).

Both cell types were grown in an incubator (CO_2_ Incubator, PBI International, Milano, Italy) at 37 °C, with 5% CO_2_ and 95% relative humidity (RU). 

0.2 cm^2^ scaffolds (5 mm diameters 0.2 mm thickness) were placed onto inserts in a 96-well plate (4.26 mm insert diameter; 3 μm membrane pore diameter, Corning Transwell Cell Culture Plate, Merck, Sigma-Aldrich, Milano, Italy) to perfectly cover them.

TEN-1 or SAOS-2 cells were seeded onto the scaffolds in the apical chamber at 8·104 cells/cm^2^ seeding density; 200 µL of PL, diluted at 1:20 in either TEN-1 (for TEN-1) or McCoy (for SAOS-2) media were placed in the basolateral chambers to simulate PL loading inside the hollow tubular scaffold; 70 µL of each medium was added in the apical chamber. Cell culture seeded directly on the insert and grown using TEN-1 or McCoy media was considered control (standard growth conditions SG). Further control was operated with cell culture seeded directly on the insert and using 200 µL PL (PL 1:20) as basolateral phase. 

After 3, 6, and 14 days, Alamar Blue test (AlamarBlue HS cell viability reagent, Invitrogen, Thermo Fisher, Monza, Italy) was performed to evaluate the metabolic activity (viability) of the cells.

#### 2.2.8. Alamar Blue Assay

At fixed times, 10% (*v*/*v*) Alamar Blue was diluted with the appropriate media and added in both the apical (70 µL) and basolateral (200 µL) chambers of each well. After 3 h incubation in dark at 37 °C, the Alamar Blue solution was collected from both the chambers and transferred in new flat wells. Each trans-well was refilled with the specific medium and left in culture again.

Alamar Blue fluorescence was recorded using a microplate reader (Microplate Reader Biotek, Synergy/HT, Fisher Scientific, Rodano (MI), Italy) with λ_ex_ = 530 nm and λ_em_ = 590 nm. 

Cytocompatibility was estimated as the ratio between the fluorescence intensities collected for the samples (scaffolds and PL 1:20 control) and the SG. 

#### 2.2.9. Immunofluorescence Analysis

Cells grown on the scaffolds or directly onto the inserts (controls) were fixed using a 3% (*w*/*v*) glutaraldehyde solution in PBS (Sigma-Aldrich, Milano, Italy) for 2 h at room temperature. The substrates were then washed three times with PBS. Extracellular matrices were stained using anti-collagen I rabbit polyclonal antibody (Thermofisher, Monza, Italy; 100 µL/sample at 10 µg/mL in PBS) to immuno-labelled collagen I from TEN-1 or bone sialoprotein antibody (Thermofisher, Monza, Italy; 100 µL/sample at 1 µg/mL in PBS) to immuno-labelled sialoprotein from SAOS-2 (24 h contact time at 4 °C). Each primary antibody was stained with ATTO 488 goat anti rabbit IgG (Sigma Aldrich, Milano, Italy), as secondary antibody (green). Cellular cytoskeleton was stained with TRICT-phalloidin (Sigma-Aldrich, Milano, Italy; 50 µL/sample at 50 µg/mL in PBS) for 40 min, in the dark. Then, each substrate was washed twice, and cell nuclei were stained with Hoechst 33258 (Sigma-Aldrich, Milano, Italy; 50 µL/sample at 1:10,000 in PBS) for 10 min in the dark. 

Scaffolds and inserts were placed onto microscope slides and imaged using a Confocal Laser Scanning Microscope (CLSM, Leica TCS SP2, Leica Microsystems, Buccinasco (MI), Italy) with (a) λ_ex_ = 346 nm and λ_em_ = 460 nm for Hoechst 33258; (b) λ_ex_ = 540 nm and λ_em_ = 565 nm for TRICT phalloidin and (c) λ_ex_ = 501 nm and λ_em_ = 523 nm for ATTO 488 goat anti rabbit IgG. The acquired images were processed with a software (Leica Microsystem, Buccinasco (MI), Italy).

#### 2.2.10. Statistical Analysis

Statistical analysis was performed using post-hoc Tukey HSD test calculator. One-way ANOVA followed by Scheffé, Bonferroni, and Holm method were considered. For the comparison of two groups, statistical significance was determined by using a two-tailed Student’s t-test method. A *p*-value ≤ 0.05 was considered statistically significant.

## 3. Results and Discussion

### 3.1. Alignment and HP-Doping Affect the Scaffold Chemical–Physical Properties

**Figure 1:**
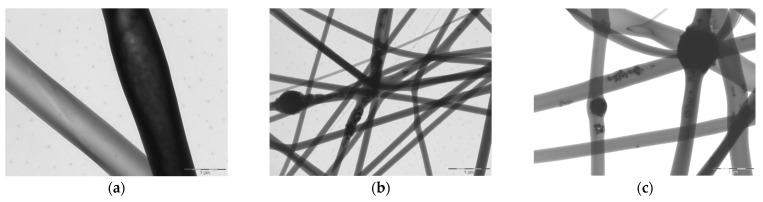
TEM microphotographs of spare electrospun fibers (scale bar 1 µm) made of B (**a**), 0.1 HP (**b**) and 0.5 HP (**c**) blends, showing the disposition and morphology of HP inclusion in individual fibers. Scale bar is 1 µm.

Figure 1 reports the TEM images of electrospun fibers made of the B, 0.1 HP and 0.5 HP blends. 

It is clearly visible that HP is embedded into the individual fibers, affecting their shape, and knots (enlargement of the nanofibers) are present due to HP nanoparticle aggregates. Also, in the knot-free portions of the HP-doped nanofibers, fine HP nanoparticles are dispersed into the polymeric matrix.

**Figure 2:**
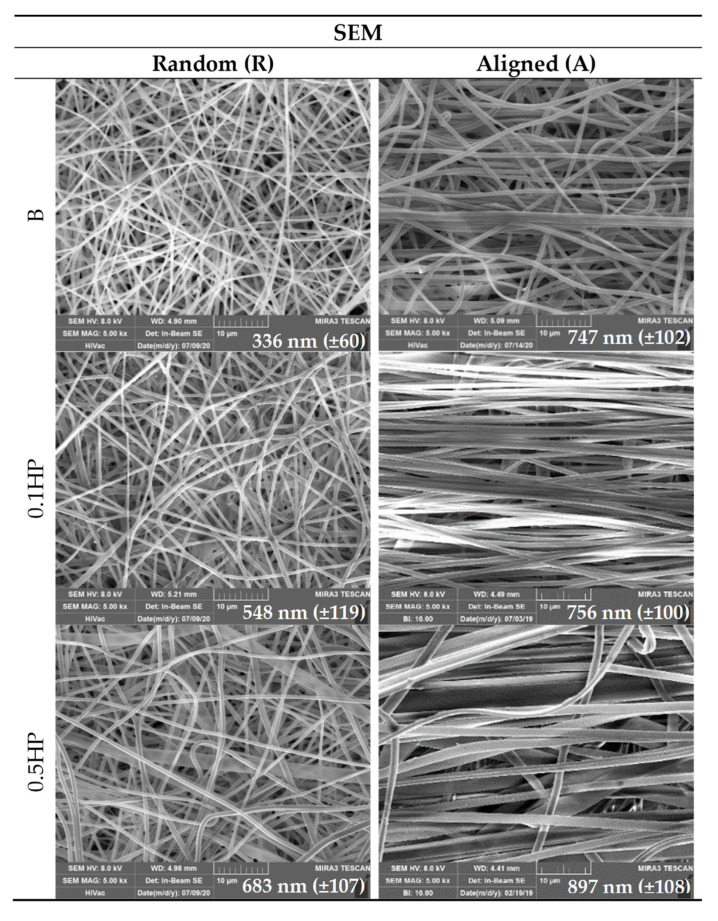
SEM (**left** column: random; **right** column: aligned) microphotographs of scaffolds: Blank; 0.1% HP and 0.5% HP. Scale bar is 10 µm. In the inset, the mean diameters of nanofibers (nm) are reported. ANOVA one-way; Scheffé test (*p* ≤ 0.01): R-B vs. R-0.5 HP, R-0.1 HP, A-B, A-0.1 HP, A-0.5 HP; R-0.1 HP vs. R-0.5 HP, A-B, A-0.1 HP, A-0.5 HP; R-0.5 HP vs. A-0.5 HP; A-B vs. A-0.5 HP; A-0.1 HP vs. A-0.5 HP (mean values ± s.d.; *n* = 60).

Figure 2 reports the SEM images of B, 0.1 HP and 0.5 HP scaffolds in aligned tubular (A) and random (R) structure. 

The surface of single nanofibers forming the scaffolds appears smooth, both for the aligned tubular and random scaffolds (SEM analysis). The average nanofiber diameters, always in the nanometric range, increase upon alignment and HP content. The knots (enlargement of the nanofibers) are clearly visible in agreement with TEM analysis, attributable to the HP nanoparticles embedded in the polymeric matrix. 

**Figure 3:**
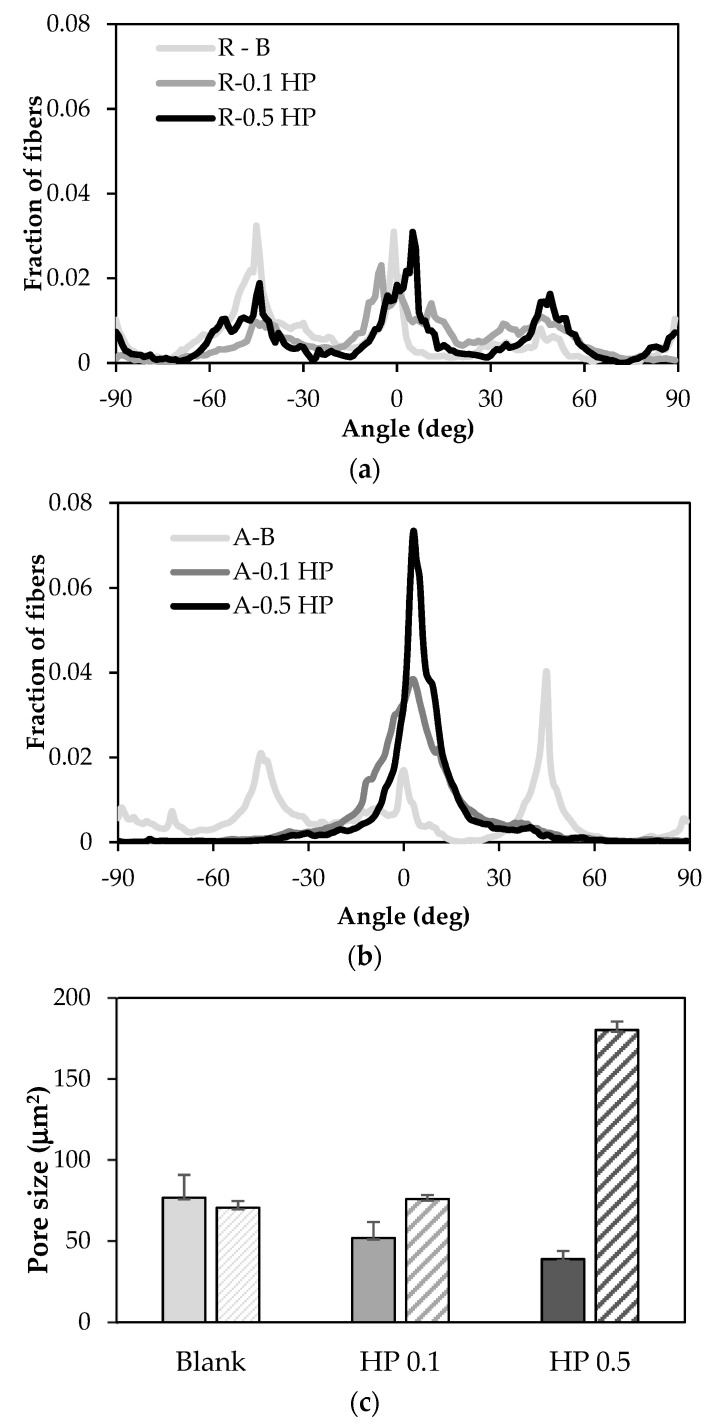
Degree of fiber orientation of blank (light grey), 0.1% HP (grey), and 0.5% HP (dark grey) scaffolds in the random (**a**) and aligned tubular (**b**) structure. The mean pore area (µm^2^) is reported in subfigure (**c**) (full color: random structure; shaded: aligned tubular). ANOVA one−way; Scheffé test (*p* ≤ 0.05): R−B vs. R−0.5% HP; A−B vs. A−0.5% HP; A−0.1% HP vs. A−0.5% HP; R−0.5% HP vs. A−0.5% HP (mean value ± s.d.; *n* = 10).

Figure 3 reports the porosity and the orientation degree of the nanofibers in the B, 0.1 HP and 0.5 HP scaffolds in aligned tubular (A) and random (R) structure, showing that both alignment and HP content affect the pore size of the scaffolds.

Porosity and anisotropy are important features, since preferentially aligned highly porous scaffolds are beneficial for cell adhesion and proliferation. Data show that, whatever the structure (random or aligned tubular), HP-free scaffolds (B) have similar porosity. Reversely, the scaffold pore size either decreases, in the random structure, or increases, in the aligned structure, upon HP content. These divergent behaviors can be attributed to the presence of knots in individual nanofibers, either improving the space filling in random scaffolds or hindering the tight packing of fibers in aligned scaffolds. Furthermore, the degree of alignment is progressively improved by HP, enhancing the surface corrugation and pattern anisotropy of the scaffold. Seemingly, HP knots are able to assist fiber collection, creating a guide to obtain alignment. 

Nonetheless, the preferential alignment of fibers is limited to the extent that it does not hinder the adaptability to “isotropic” cells cultures and does not spoil the felting of the scaffold.

**Figure 4:**
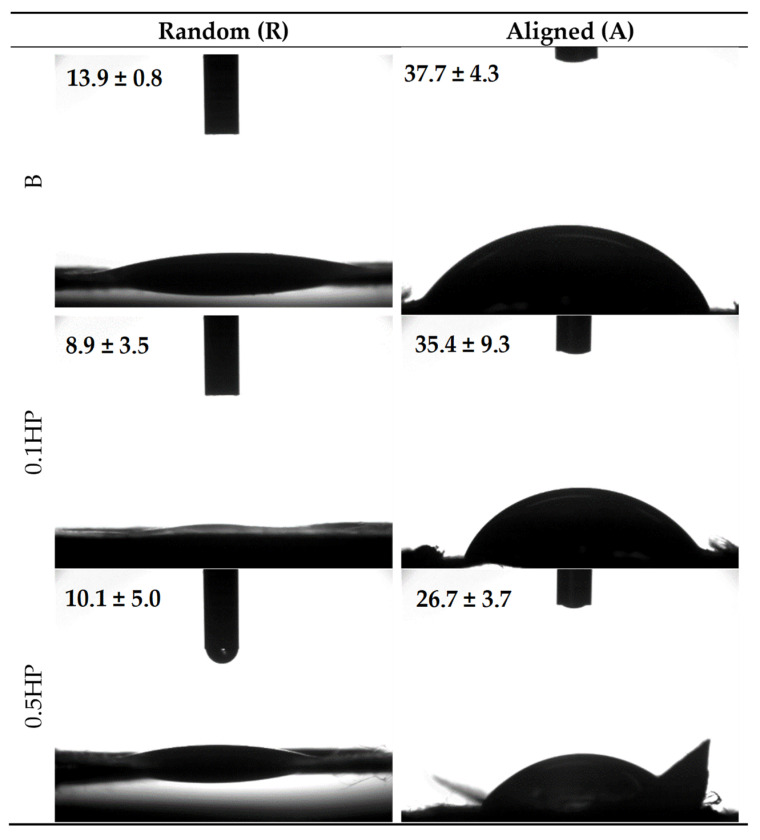
Images of the buffer drop onto the scaffold surface after 1000 ms. In each image, the value of the contact angle is reported (mean values ± s.d.; *n* = 3) (needle diameter = 0.405 mm).

Figure 4 shows the shape and the contact angles for a 0.4 µL buffer drop released onto B, 0.1 HP, and 0.5 HP scaffolds, in the aligned tubular (A) and random (R) structures. It can be seen that the fibers alignment governs the wettability of the scaffolds, independent of the variable HP-doping, and despite the same polymeric matrix composition.

In fact, all the random scaffolds show similar lower contact angles, and consequently are more wettable than aligned scaffolds, displaying similar higher contact angles. It appears that the surface organization either allows or prevents the water spreading onto the scaffold surface. This could be ascribed to the interfiber spacing in the aligned nanofibers leading to a capillary-like force parallel to the fiber orientation, while preventing water spreading in the cross direction. In randomly oriented scaffolds, the boundary-induced forces induced by adjacent nanofibers are randomly directed without any influence on hydration [26]. 

**Figure 5:**
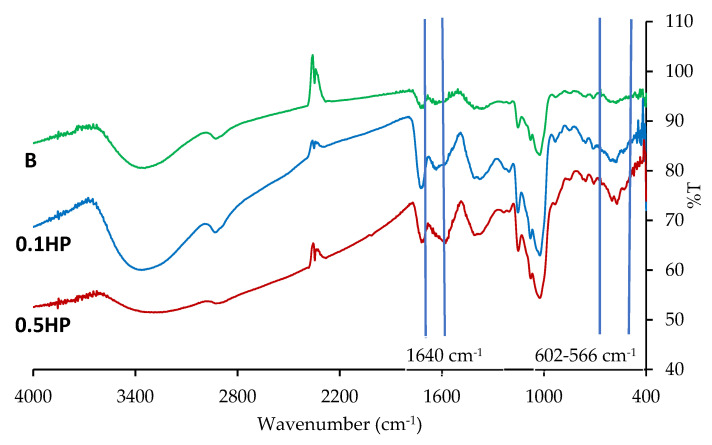
FT-IR profiles of the blank, 0.1 HP, and 0.5 HP scaffolds after cross-linking.

Figure 5 reports the FT-IR profiles recorded for the dry random scaffolds (R-B, R-0.1 HP, R-0.5 HP), all presenting similar patterns typical of polysaccharides. The peak in the region around 1640 cm^−1^, typical of the Amide I band, is likely due to covalent bonds occurring between chitosan amino groups and carboxylic moieties of citric acid [14] occurring in the crosslinking stage of scaffold preparation protocol. In the HP-doped scaffolds, characteristic vibrational bands at 602 cm^−1^ and 566 cm^−1^, arise from the HP phosphate groups [18], as expected.

### 3.2. Alignment and HP-Doping Modulate the Scaffold Mechanical Properties on the Macroscopic and Molecular Scales

**Figure 6:**
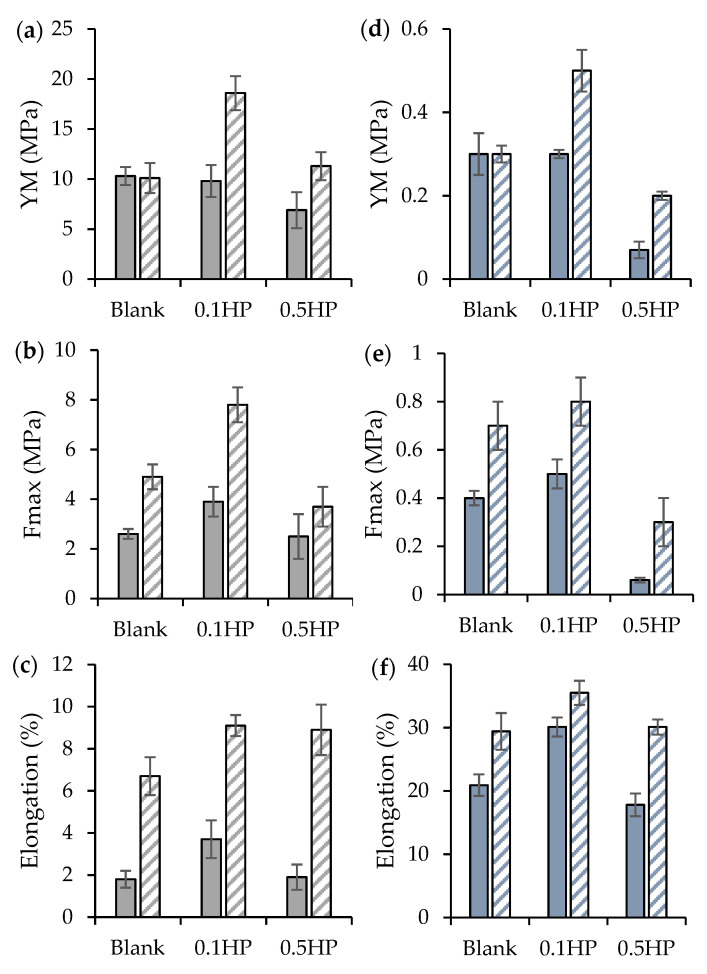
Mechanical properties of random (R, full color) and tubular aligned (A, shaded) scaffolds in the dry (top row) and hydrated (bottom row) states: (**a**,**d**): Young modulus (YM); (**b**,**e**): Fmax; (**c**,**f**): elongation at break (mean values ± s.d.; *n* = 4).

Figure 6 reports the mechanical properties (a, d: Young modulus (YM); b, e: force-at-break, Fmax; c, f: elongation) of both random (R) and tubular aligned (A) scaffolds in the dry (a, b, c) and hydrated (d, e, f) states.

As a general trend, all aligned samples show up more rigid (in the elastic regime, higher YM) than their random counterparts, and able to stand higher stress and larger deformation before breaking. On the other hand, it appears that moderate doping with HP (0.1 HP samples, both R and A) is significatively the most beneficial for the mechanical performance of scaffolds, strengthening their structure. Reversely, excessive HP addition (0.5 HP) is seemingly detrimental for the polymer chains entanglement, thus weakening the scaffolds structure [27,28].

**Figure 7:**
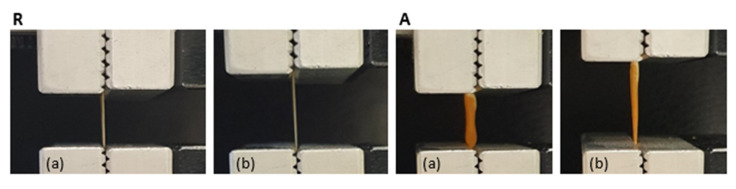
Macroscopic pictures of random (R) and tubular aligned (A) scaffolds at relaxed (**a**) and elongated state (**b**).

Figure 7 reports the macroscopic pictures of both random (R) and tubular aligned (A) scaffolds at relaxed (a) and elongated state (b). Upon hydration, samples become roughly three-fold less rigid than in their dry state, and stand much longer elongation (in the range 20–35% of their length at rest) before breaking. 

**Figure 8:**
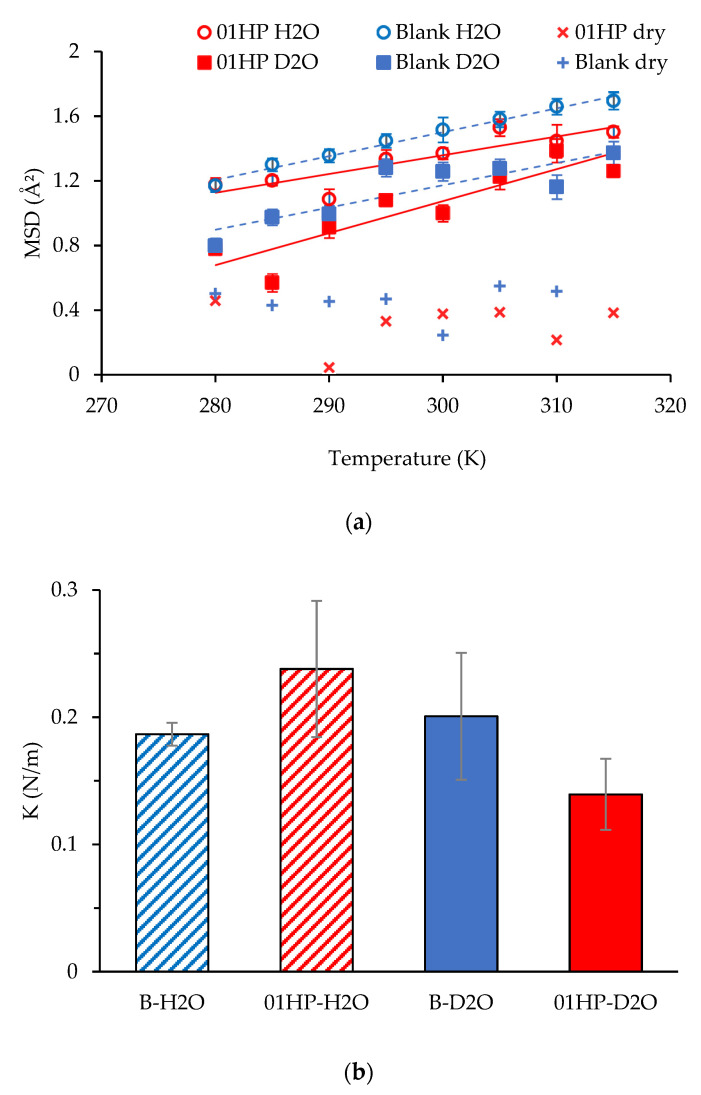
Panel (**a**): MSD values (for B and 0.1 HP scaffolds, dry (crosses) and hydrated in either H_2_O (blue) or D_2_O (red). Panel (**b**): local resilience determined for HP-free or HP-doped scaffolds (mean values ± s.d.; *n* = 4).

As seen in Figure 8, panel a, the scaffold local mobility is also released upon wetting with either H_2_O or D_2_O. Measurements performed in D_2_O (squares), enhancing the visibility of un-exchangeable hydrogens, show that the MSD of molecules is more than doubled in the wet condition, an extent that is further enlarged if all hydrogens are accounted for (H_2_O wetting, circles). Seemingly, the un-exchangeable hydrogens of the HP-free scaffold are more resilient (higher K value, Figure 8, panel b) than the ones in the HP-doped scaffold, where the intercalation of HP nanoparticles might locally impede compaction of the polysaccharide chains.

Thus, HP-doping could promote the mechanics of cell growth by relieving the local stiffness of the scaffold. On the other hand, it seems that the macroscopic mechanics of scaffolds are governed by their large-scale structure.

Notably, and importantly, the systems display mechanical properties, both elasticity and force-at-break, similar to those of the native tendon. In fact, typically, the ultimate tensile strength of native tendon and ligament ranges from 5 to 100 MPa with a strain of failure 10–15% and a Young’s modulus from 20 to 1200 MPa [6].

Assuming the muscle-specific tension of 0.3 MPa, the stress generating potential of a tendon is estimated around 30 MPa. The tendon strain ranges between 2 and 5% at the maximum tendon stress [29].

Moreover, the tendon mechanical properties are directly related to ECM hierarchical structure mimicked using the tubular aligned conformation and the polymer matrix doping with HP nanoparticles—0.1% HP proved to be suitable to reinforce the structure; in fact, it was demonstrated that the mechanical properties can be remarkably improved by adding the optimum amount of nanoparticles, due to their fine dispersion. On the other hand, higher concentrations could affect nanoparticle dispersion and particle inhomogeneity, and agglomeration lead to structural alterations and, consequently, a reduction of the mechanical properties [30].

### 3.3. HP-Doped Scaffolds Can Control the Release of Bioenhancer Proteins

**Figure 9:**
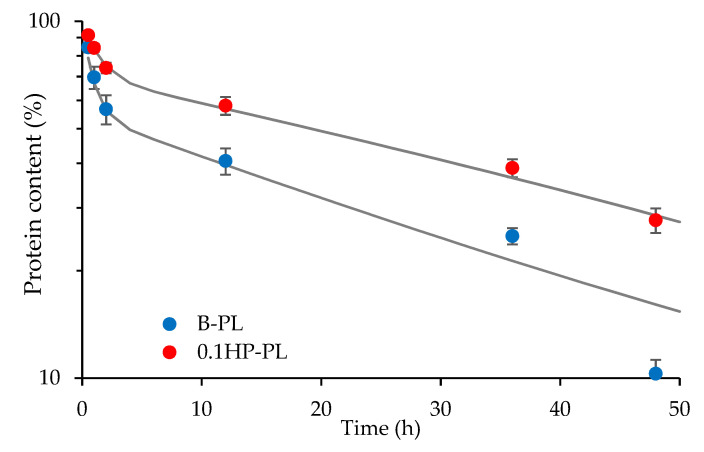
Profile of protein release from tubular aligned scaffolds in the trans-well system configuration. Platelet lysate (PL) diluted 1:20 in HBSS was placed in the lower chamber. B (blue dots) and 0.1 HP (red dots) scaffolds were placed over the insert in the upper chamber, see text (mean values ± s.d.; *n* = 4). The fitting curves are drawn in grey.

Blank and HP-doped tubular aligned scaffolds, B and 0.1 HP, were tested for their capability to control the permeation of proteins of the platelet lysate (PL). In fact, the tubular structure was intended to achieve controlled and prolonged GF action over time, by loading PL into the inner cavity. As reported in Figure 9, both scaffolds allowed for PL proteins’ diffusion across the scaffold walls over quite long times. In both cases, a fast permeation, in the first 2 h, is followed by a slower one, reaching substantial protein release in 48 h. Data can be fitted by a double exponential time-decay, as in the presence of fast- and slow-diffusing proteins in PL. Both components suggest that diffusion is slower in the HP-doped scaffold than in the blank one, although the pore size is similar (Figure 3).

Besides a possible difference in the total pore area, the increased scaffold hindrance could be due to interactions occurring between HP nanoparticles and PL proteins. In fact, as reported in the literature, the carboxylic groups of proteins are able to interact with Ca^2+^ ions, thus bridging to the phosphate groups of HP [31]. As a result, controlled protein permeation through the scaffold wall might be further slowed down by the presence of HP and the PL activity be prolonged.

### 3.4. HP-Doped Tubular Scaffolds Sustain Prolonged Bio-Enhanced Cell Proliferation

**Figure 10:**
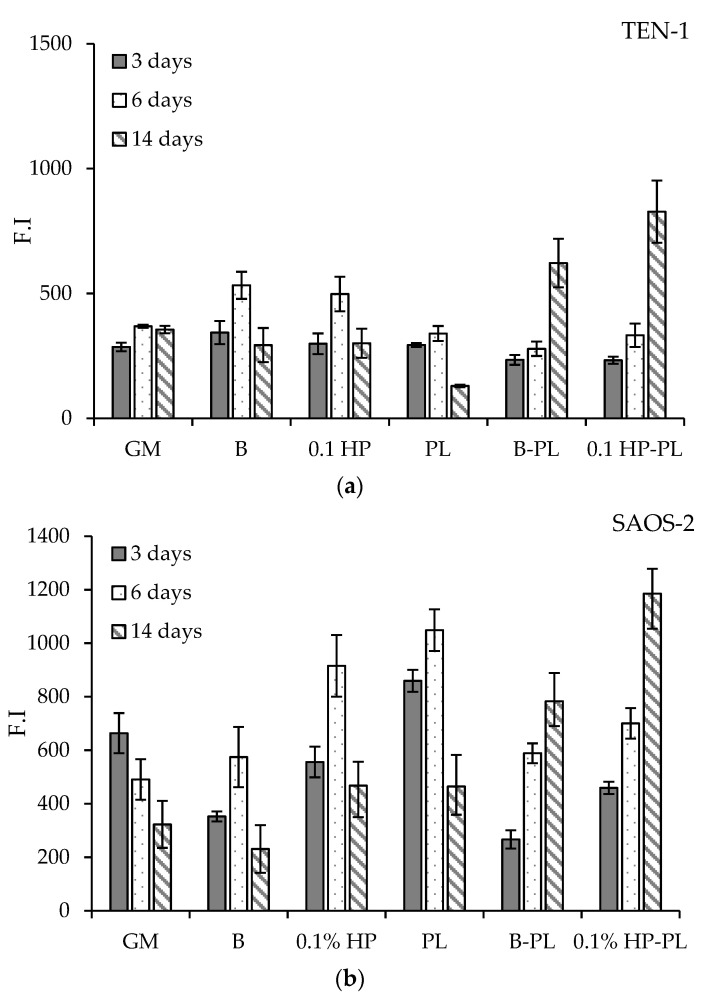
Viability (as fluorescence intensity, F.I.) of TEN-1 (**a**) and SAOS-2 (**b**) at 3, 6, and 14 days from seeding onto blank and HP-doped aligned scaffolds, in absence and in presence of PL (mean values ± e.s.; *n* = 8). Comparison is also made with cells seeded without any scaffold, in either GM or PL (see text).

In view of the application of aligned tubular scaffolds in the repair of the osteo–tendon interface, two cell lines were selected as representatives for tendon and bone cells, namely TEN-1 and SAOS-2, and grown onto the aligned scaffolds (B, 0.1 HP). Their metabolic activity, directly related to their viability, was assessed at increasing time-delays over 14 days from seeding and is reported in Figure 10, in terms of fluorescence intensity, F.I. In parallel experiments, cells were also grown in the absence of any scaffold, for comparison, as hereafter detailed.

Tendon TEN-1 cells were able to proliferate in the GM (growth medium) over the entire time interval of observation, although showing a progressive decrease in viability (Figure 10, top panel, GM block). The TEN-1 cells seeded on the aligned scaffolds showed the same viability in GM; the presence of HP improved their vitality at intermediate times. As expected, addition of PL to the growth medium enhanced cell viability, with a boosting action that is nonetheless mainly restrained to short and intermediate times (1 and 3 days). Conversely and notably, the synergic effect of the scaffolds was seen, protracting and reinforcing the action of PL on TEN-1 vitality to longer times, still increasing at 14 days from seeding. This effect can be attributed to the modulation and control of PL diffusion operated by the scaffold. Further improvement is seen when HP is included in the scaffold fibers, thus sustaining PL-loaded, HP-doped aligned tubular scaffolds as profitable supports for tendon repair.

Similar results were obtained for bone SAOS-2 cells (Figure 10, bottom panel). Seeding on scaffolds did not hinder cell viability in GM; rather, some increase was observed at intermediate times. Notably, the controlling effect played by scaffolds on PL diffusion was particularly effective on SAOS-2, prolonging and promoting cell viability at long times, with a positive contribution played by HP-doping of the scaffold fibers.

The positive results obtained on both TEN-1and SAOS-2 cells thus endorse PL-loaded HP-doped aligned tubular scaffolds as a strategic platform for osteo–tendon interface reconstruction, constituting a suitable biologically-enhanced mechanical substrate to promote both hard and soft tissue proliferation following surgical treatment.

Moreover, it has been noted that HP is effective in skin healing due to its affinity with collagen, and that the progressive bio-dissolution of HP into its Ca^2+^ and PO_4_^3−^ ions constituents sustains collagen neo-formation [32].

### 3.5. Anisotropy, HP-Doping, and PL-Bioenhancing Synergistically Aid Proper Morphology and ECM Production of Tendon and Bone Cells

**Figure 11:**
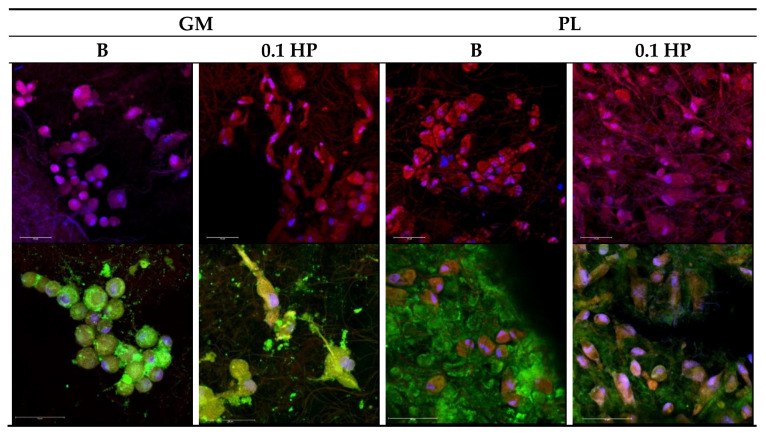
CLSM images of TEN-1 grown onto the aligned scaffolds (B and 0.1 HP) after 14 days with or without platelet lysate (PL) (PL 1:20 dilution in growth medium). Actin stained in red, nuclei stained in blue and collagen I stained in green (magnifications: 40× upper row or 63× lower row).

**Figure 12:**
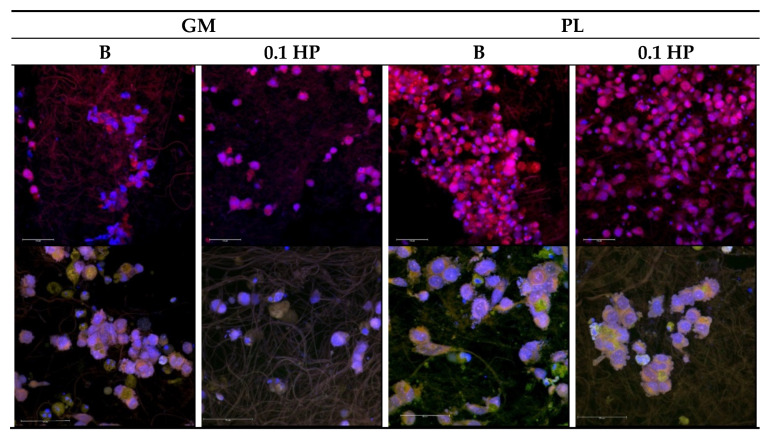
CLSM images of SAOS-2 grown onto the aligned scaffolds (B and 0.1 HP) after 14 days with or without platelet lysate (PL) (PL 1:20 dilution in growth medium, GM). Actin stained in red, nuclei stained in blue and sialoprotein stained in green (magnifications: 40× upper row or 63× lower row).

Functional repair of osteo–tendon interface requires that, beyond the tested viability and growth, cells of both bone and tendon tissue develop with the proper morphology and ability to produce a suitable extracellular matrix. Figure 11 and Figure 12 report the CLSM images of the TEN-1 and SAOS-2 cells (respectively) seeded onto the HP-free and HP-doped aligned scaffolds, with and without PL-bioenhancement, at 14 days from seeding; that is, after the full period of cell viability observation (see Figure 10).

As expected, tendon TEN-1 cells could proliferate in all tested conditions (Figure 11, upper row); nonetheless, in the absence of PL bioenhancer, their growth onto both HP-free and HP-doped aligned scaffolds resulted in clusters of cells with a round shape, quite far from the spindle morphology typical of the normal tenocytes. Similarly, tendon TEN-1 cells could produce collagen-1 (green signal in Figure 11 bottom row) in all tested conditions; still, its correct localization occurs only in the presence of PL. In the absence of PL, collagen appears confined in the perinuclear intracellular region. On the contrary, the presence of PL was able to induce collagen secretion, giving a uniform extracellular matrix (green background, Figure 11) for tenocytes grown onto both HP-free (B) and HP-doped (0.1 HP) scaffolds. On the other hand, while the presence of PL was fundamental to enhancing cell proliferation onto both HP-free and HP-doped scaffolds, only the simultaneous presence of PL-bioenhancing and HP-doping enabled the tenocytes to reach confluency onto the scaffold with the correct morphology. In this respect, the local softening of the polymer matrix provided by the presence of HP may help in structural adaptation to proper cell growth.

For bone cells (Figure 12), as expected from viability data, the presence of PL was needed for scaffolds to sustain SAOS-2 growth and proliferation, and only HP-doping allowed for wide spreading of the cells all over the scaffold surface, leading to cell confluency. Again, the synergistic actions of PL-loading and HP-doping show up in the capability of bone cells to properly proliferate onto the aligned scaffold (Figure 12, upper row) and produce a well-developed extracellular matrix, here identified through sialoproteins staining (Figure 12, bottom row), and richly decorated with extracellular vesicles.

## 4. Conclusions

Electrospinning was successfully used to prepare polysaccharide-based preferentially-aligned HP-doped tubular scaffolds aimed at the functional reparation of tendon injury and the reconstitution of osteo–tendon interfaces. The tubular structure, with cross- sections in the mm range, was intended to allow for extemporary loading of hemoderivatives, like PL, concomitant with the surgical intervention, while performing controlled and prolonged release of bioenhancer molecules to support tissue repair in the post-surgery period. Notably, and importantly, the scaffolds display mechanical properties similar to those of the native tendon, and HP-doping confers local softness to the polysaccharide matrix. Controlled protein permeation was verified to occur and to promote and sustain prolonged growth of both tendon and bone cells, with appropriate morphological features and proper ECM production.

## Figures and Tables

**Table 1:** Scaffolds quali-quantitative composition.

Scaffold	Pullulan % (*w*/*w*)	Chitosan % (*w*/*w*)	Citric Acid % (*w*/*w*)	HP % (*w*/*w*)
Blank	66.8	16.6	16.6	
HP0.1	66.3	16.5	16.5	0.7
HP0.5	64.6	16.1	16.1	3.2

## Data Availability

Data available on request.

## Appendix A

Backscattering experiments were performed on the B and 0.1 HP scaffolds, in the random structure, in the dry and D_2_O or H_2_O hydrated conditions. Rectangular dried cuts (3 × 5 cm^2^) of the scaffolds were enclosed in air-tight aluminum flat cells of known mass, weighted and located in place for measurement. Subsequently, cuts were extracted from the aluminum cells, carefully dried and desiccated, and then fully immersed in D_2_O. After draining the excess D_2_O, scaffolds were enclosed again in the original air-tight aluminum cells, then weighted and measured. The same procedure was repeated for H2O-hydration. Experiments were performed on the thermal (λ = 2.23 Å) high-energy resolution backscattering spectrometer IN13 (Institut Laue-Langevin, ILL, Grenoble, France), in the temperature range 280 < T < 315 K. The elastic neutron scattering intensity, S(q, ω = 0), collected at different scattering angles θ, is reported as a function of q = 2π/λ sin(θ/2). In the low-q region, the q-dependence of S(q, ω = 0) is given, according to the Gaussian approximation, by: S(q, ω = 0) = I_0_ exp(−<u^2^> q^2^/6) where I_0_ is a constant and <u^2^> is the mean-square amplitude of atomic displacements (MSD). Thus, from the slope of the semi-logarithmic plot of the incoherent scattering function vs q^2^, the mean square displacements (MSD) for a given T is extracted. The temperature variation of the MSDs can be interpreted in terms of an empirical effective force constant <k′>, called resilience by Zaccai [25]. It describes the rigidity or resilience of the macromolecules. The effective average force constant for sample dynamics, <k′>, can then be calculated from the slope of MSD as a function of temperature, by applying a quasi-harmonic approximation: <k′> = 0.00276 (d<u^2^>/dT).
